# Immune-Modulatory Mechanism of Compound Yeast Culture in the Liver of Weaned Lambs

**DOI:** 10.3390/ani16010104

**Published:** 2025-12-30

**Authors:** Chenlu Li, Hui Bai, Pengxiang Bai, Chenxue Zhang, Yuan Wang, Dacheng Liu, Hui Chen

**Affiliations:** 1 College of Veterinary Medicine, Inner Mongolia Agricultural University, Hohhot 010018, China; lclimau2022@163.com (C.L.); baihui101214@163.com (H.B.); bpx2021@126.com (P.B.); x408337660@163.com (C.Z.); wangyy4801@163.com (Y.W.); 2 Key Laboratory of Clinical Diagnosis and Treatment Techniques for Animal Disease, Ministry of Agriculture, Hohhot 010019, China

**Keywords:** compound yeast culture, weaned lambs, hepatic immune function, transcriptomics

## Abstract

Compound yeast culture (CYC) is known to enhance animal health, but its effects on hepatic immune function are unclear. This study systematically examined CYC’s regulatory effects on the liver of weaned lambs using transcriptomics and integrative bioinformatics. Ten lambs were randomly assigned to a control diet or a basal diet supplemented with 30 g/d per head of *Saccharomyces cerevisiae* and *Kluyveromyces marxianus* co-culture (CYC group) for 42 days. Histological analysis showed that CYC improved hepatocyte arrangement and sinusoidal integrity, suggesting enhanced hepatic tissue stability. Cytokine analysis revealed CYC significantly increased IL-6 and IL-1β while reducing IL-10, TGF-β1, *TNF-α*, and *CXCL9*, indicating a bidirectional modulation of the immune response. Additionally, CYC enhanced antioxidant defenses by increasing T-SOD, GSH-Px, and T-AOC activities and decreasing MDA content. Transcriptomic sequencing indicated that CYC reshaped hepatic gene expression. Upregulated genes were enriched in immune-regulatory and structural pathways, including PI3K-AKT signaling, ECM–receptor interactions, Toll-like receptor pathways, and cell adhesion molecules. Protein-level validation further confirmed activation of PI3K and AKTAKT phosphorylation with limited engagement of NF-κB signaling. Conversely, downregulated genes were mainly associated with oxidative stress and energy metabolism, such as ROS-related pathways and MAPK signaling. WGCNA identified key hub genes (*PTPRC*, *CD86*, and *ITGAV*), which correlate with pro-inflammatory factors and participate in immune recognition, T-cell activation, and cell adhesion. These data suggest that CYC promotes hepatic immune homeostasis by enhancing immune signaling, stabilizing tissue architecture, and modulating oxidative stress/metabolic processes. This study provides mechanistic insights into CYC’s regulation of liver immune function and supports its targeted application as a functional feed additive for ruminants.

## Simple Summary

Weaning is a stressful transition period for lambs, often associated with oxidative stress, immune dysregulation, and compromised liver function. This study investigated whether supplementation of the daily ration with a compound yeast culture (CYC), a co-culture of *Saccharomyces cerevisiae* and *Kluyveromyces marxianus*, could support hepatic function in weaned lambs. CYC supplementation improved liver tissue structure, enhanced antioxidant defenses by increasing T-SOD, GSH-Px, and T-AOC activities while reducing lipid peroxidation product MDA, and balanced immune responses by modulating pro- and anti-inflammatory cytokine levels. Molecular analyses revealed that CYC upregulated PI3K–AKT signaling, contributing to maintenance of liver structure and immune homeostasis. Collectively, these findings indicate that inclusion of CYC in the daily ration is an effective nutritional strategy to support liver integrity, reinforce immune and oxidative balance, and help lambs better cope with weaning-associated challenges.

## 1. Introduction

In ruminant husbandry, weaning is a pivotal stage in lambs, and studies have shown that weaning stress affects lambs’ feeding behavior and physiological state, thereby impairing their overall growth and development [1]. Early weaning induces severe stress in lambs, directly affecting liver function by increasing fatty acid oxidation, altering immune activity, and elevating oxidative stress [2]. Consistently, recent studies have shown that post-weaning stress modulates hepatic antioxidant enzyme activity and ruminal barrier function [3]. These changes ultimately hinder the development of ruminants. Environmental shifts and dietary changes after weaning disrupt physiological homeostasis, causing both metabolic and immunological stress.

The liver is a vital organ in ruminants, functioning in both metabolic regulation and immune defense, and is essential for maintaining systemic homeostasis. It is primarily responsible for carbohydrate and lipid metabolism [4], toxin elimination [5], bile synthesis, and other physiological processes, while also acting as an immune organ involved in antigen recognition, inflammatory regulation, and acute phase responses [6]. Under modern intensive farming, ruminants are chronically exposed to stressors such as harsh environments and pathogenic microbes. These stressors increase hepatic burden, leading to metabolic disorders, chronic inflammation, and tissue damage [7], ultimately compromising animal health and productivity. Therefore, developing safe and effective nutritional strategies to strengthen hepatic immune and anti-inflammatory capacity has become a major focus in animal nutrition and health research.

In recent years, functional microecological preparations have garnered increasing attention in ruminant health regulation due to their environmentally friendly, safe, and residue-free properties [8]. Yeast cultures, widely used as dietary additives for ruminants, have been shown to improve production performance, maintain gastrointestinal microbiota homeostasis [9], enhance immune function, increase antioxidant capacity [10], and reduce weaning-induced diarrhea. Among them, compound yeast culture (CYC) is obtained by co-fermentation of multiple yeast strains (*Saccharomyces cerevisiae* and *Kluyveromyces marxianus*) as previously described by our group [11]. CYC contains β-glucans, manno-oligosaccharides, nucleotides, and bioactive small molecules, conferring multiple biological effects such as microecological regulation, immune enhancement, and antioxidant activity [10,12]. Previous studies have shown that CYC improves growth performance and immune capacity by enhancing rumen fermentation, promoting nutrient absorption, and strengthening intestinal barrier function [9,13]. Despite growing evidence for its efficacy in animal production [10,14], the mechanisms by which CYC regulates liver immunity and antioxidant capacity in weaned lambs remain poorly understood [12]. In particular, evidence at the cellular signaling level is limited, and it is unclear whether CYC modulates the hepatic immune environment and antioxidant defense through specific immune- and redox-related pathways [15].

In this study, we used weaned lambs as a model to investigate the effects of dietary CYC supplementation on the hepatic immune microenvironment and oxidative stress. By integrating histological analyses, inflammatory cytokine profiling, transcriptome sequencing, and Weighted Gene Co-expression Network Analysis (WGCNA), we identified key immune-related pathways modulated by CYC, including Toll-like receptor (TLR) signaling and cell adhesion molecules (CAMs). Protein-level validation using phosphorylation-specific Western blotting further highlighted the PI3K-AKT and NF-κB signaling axes as central nodes, elucidating the mechanisms by which CYC regulates hepatic immune activation and redox homeostasis during the post-weaning period. These results advance our understanding of CYC-mediated hepatoprotection in ruminants, reveal its molecular targets for immune and oxidative regulation, and provide a foundation for microbiome-targeted strategies to enhance liver health and immunity in young ruminants.

## 2. Materials and Methods

### 2.1. Preparation of Compound Yeast Culture

Based on preliminary work, *Saccharomyces cerevisiae* and *Kluyveromyces marxianus* strains with strong fermentation capacity were isolated from naturally fermented mare’s milk. These strains were used as fermentation agents with a solid-state medium consisting of corn germ meal (28%), spray-dried corn bran (12%), bran (12%), rice bran (10%), soybean meal (10%), maize (10%), cottonseed meal (10%), and wheat middlings (8%) [9,13].

The strains were mixed in a 1:1 ratio (3 × 10^8^ CFU/g live cells) and inoculated at 8% of the substrate wet weight. Sterile water was added to adjust the moisture content to ~40%. The mixture was homogenized and fermented aerobically at 30 °C for 72 h [14]. The compound yeast culture contained ≥ 18% crude protein, ≤12% moisture, ≤9% crude ash, ≥0.5% mannan, and a viable yeast count of ≥10^6^ CFU/g. The nutrient compositions of the CYC are presented in Table S1 [9,14].

### 2.2. Experimental Design and Animal Diets

Ten healthy, weaned male lambs (2 months old; 23.5 ± 2.85 kg) with uniform body condition were randomly allocated to two groups: control and CYC, with five lambs per group. The control group received a basal total mixed ration (TMR), while the CYC group received a basal TMR diet supplemented with 30 g CYC per lamb per day (1.5% of dietary dry matter). The trial lasted 42 days, including a 7-day adaptation period and a 35-day main experiment. Lambs were housed individually under natural ventilation with ad libitum access to feed and water. Feeding was provided twice daily at 08:00 and 17:00. Diets were formulated according to NRC (2007) standards [16] to meet maintenance and growth requirements, providing 15% crude protein, 11 MJ/kg metabolizable energy, and a Ca:P ratio of 1.8. Apart from CYC supplementation, all other nutritional components were identical between groups [12].

### 2.3. Sample Collection

At the end of the trial, sheep were fasted for 12 h to minimize interference from gastric contents during sampling. Animals were humanely euthanized by intravenously injecting sodium pentobarbital according to standard procedures, followed by rapid post-mortem collection of liver tissue. Fresh liver samples were placed in pre-chilled sterile phosphate-buffered saline (PBS) and rinsed repeatedly to remove blood and impurities. The cleaned tissue was minced, aliquoted into sterile cryogenic tubes, snap-frozen in liquid nitrogen, and stored at −80 °C for transcriptome sequencing and quantitative reverse transcription (qRT)-PCR. In addition, a 2 × 2 cm liver block was fixed in 4% paraformaldehyde overnight at 4 °C for histological examination.

### 2.4. Observation of the Histological Structure of Liver Tissue

Liver tissue blocks were collected with sterile surgical scissors and washed with pre-chilled PBS (pH 7.2) to remove residual blood. Approximately 2 cm^2^ of tissue, including portions of the left, right, and caudate lobes, was fixed in 4% paraformaldehyde overnight. Fixed tissue was subjected to graded dehydration, paraffin embedding, and sectioned into 8 μm slices. Sections were stained with routine hematoxylin and eosin (H&E). Six sections per animal were prepared, each in triplicate. Morphological features, including hepatocyte arrangement, central veins, and sinusoidal structures, were examined using Image-Pro Plus 6.0 (Media Cybernetics, Bethesda, MD, USA).

### 2.5. Determination of Hepatic Immune and Antioxidant Parameters

#### 2.5.1. Determination of Hepatic Immune Cytokine Levels by ELISA

To assess the regulatory effects of CYC on hepatic immune function in lambs, immune factor levels in liver tissue were determined using enzyme-linked immunosorbent assay (ELISA). About 1 g of liver tissue was homogenized in pre-chilled PBS, centrifuged at 3000 rpm for 10 min at 4 °C, and the supernatant was collected. The concentrations of interleukin-1β (IL-1β), interleukin-6 (IL-6), interleukin-10 (IL-10), tumor necrosis factor-α (TNF-α), and transforming growth factor-β1 (TGF-β1) were measured using ELISA kits (Ruixin Biological, Quanzhou, China). All assays were performed strictly according to the manufacturer’s instructions.

#### 2.5.2. Detection of Relative mRNA Expression Levels of Hepatic Immune Cytokine Levels in Liver Tissue by qRT-PCR

Trizol (TIANGEN., Ltd., Shanghai, China) was used to extract RNA from liver tissues. Subsequently, a Nanodrop 2000 spectrophotometer (Thermo Fisher Scientific, Waltham, MA, USA) was employed to assess the RNA quality. An OD260/OD280 ratio ranging from 1.8 to 2.0 indicated that the RNA was sufficiently pure for further use. Total RNA from the liver tissue was subsequently reverse-transcribed into cDNA, and analyzed by qRT-PCR to determine the mRNA expression of *IL-1β*, *IL-6*, *TNF-α*, and C-X-C motif chemokine ligand 9 (CXCL9). Primers were designed based on target gene sequences obtained from the NCBI database and synthesised by Sangon Biotech Co., Ltd. (Shanghai, China). Relative expression levels were calculated using the 2^−ΔΔCt^ method [17], with GAPDH as the internal reference gene (qRT-PCR kit from Novazene, Nanjing, China). The primer sequences used in this study are provided in Table 1.

#### 2.5.3. Determination of Oxidative Stress–Related Parameters

Hepatic oxidative stress parameters were determined using commercial biochemical assay kits according to the manufacturers’ instructions. Liver samples (~0.1 g) were homogenized in nine volumes of ice-cold physiological saline to prepare a 10% (*w*/*v*) homogenate. The homogenate was centrifuged at 2500× *g* for 10 min at 4 °C, and the supernatant was collected for analysis.

Total superoxide dismutase (T-SOD, Cat. No. A001-3-2), glutathione peroxidase (GSH-PX, Cat. No. A005-1-2), total antioxidant capacity (T-AOC, Cat. No. A015-3-1), and malondialdehyde (MDA, Cat. No. A003-1-2) were measured using kits from Nanjing Jiancheng Bioengineering Institute (Nanjing, China). Protein concentration of each sample was determined using a BCA assay kit (Beyotime, Shanghai, China), and the activities of T-SOD, GSH-PX, T-AOC, as well as the MDA content, were normalized to protein concentration.

### 2.6. Transcriptome Sequencing

#### 2.6.1. RNA Extraction

Total RNA was isolated from lamb liver tissue with TRIzol^®^ reagent following the protocol provided by the manufacturer. The RNA integrity was evaluated using an Agilent 5300 Bioanalyzer, while its concentration was determined with a NanoDrop ND-2000 spectrophotometer (NanoDrop Technologies, Wilmington, DE, USA). For subsequent library preparation, only RNA samples fulfilling the following quality requirements were included: an OD260/280 ratio between 1.8 and 2.2, OD260/230 not less than 2.0, an RNA quality number (RQN) of at least 6.5, a 28S:18S ribosomal RNA ratio of 1.0 or higher, and a total RNA yield exceeding 1 μg.

#### 2.6.2. Library Preparation and Sequencing

RNA purification, reverse transcription, library preparation, and sequencing were conducted by the Majorbio Cloud Platform (Shanghai Majorbio Biotechnology Co., Ltd., Shanghai, China; www.majorbio.com, accessed on 10 March 2025) [18]. Library preparation was performed with the Illumina^®^ Stranded mRNA Prep Ligation Kit (Illumina, San Diego, CA, USA), using 1 μg of total RNA per sample. Poly(A)-tailed mRNA was first enriched using oligo(dT) magnetic beads and then fragmented. Double-stranded cDNA was synthesised using random hexamer primers. Subsequent steps included end repair, phosphorylation, and adapter ligation. After magnetic bead purification, DNA fragments of 300–400 bp were selected and amplified by PCR for 10–15 cycles. The amplified libraries were quantified with a Qubit 4.0 fluorometer and sequenced using the NovaSeq Reagent Kit on an Illumina NovaSeq X Plus platform with paired-end 150 bp reads (PE150).

#### 2.6.3. Quality Control and Comparison Analysis

Raw sequencing data were processed with fastp (default parameters) for quality control and adapter removal, generating high-quality clean reads. The clean reads were then aligned to the sheep reference genome [19] using HISAT2 in directional mode [20]. Alignment results were further processed with StringTie [21] to assemble and quantify transcripts based on the reference genome.

#### 2.6.4. Differential Expression Analysis and Functional Enrichment

Differential expression analysis was performed using DESeq2 on raw read counts from RSEM [22]. DESeq2 models gene counts with a negative binomial distribution and uses Wald tests to assess expression differences between groups [23]. *p*-values were adjusted by the Benjamini–Hochberg method, and genes with |log_2_ fold change| ≥ 1 and FDR < 0.05 were considered differentially expressed. Identified differentially expressed genes (DEGs) were subjected to GO and KEGG enrichment analyses using KOBAS (version 3.0), with Bonferroni-corrected *p*-value < 0.05 considered significant.

#### 2.6.5. Validation of Transcriptome Differential Expression Genes

To confirm the reliability of DEGs identified via transcriptome sequencing, representative up and downregulated genes were selected for validation by qRT-PCR. RNA samples were the same as those used for transcriptome sequencing. cDNA was synthesized using a reverse transcription kit according to the manufacturer’s instructions. Each sample was analyzed in triplicate. GAPDH was used as the internal control, and relative expression levels were calculated using the 2^−ΔΔCt^ method. Primers for DEG validation were designed based on target gene sequences obtained from the NCBI database. All primer sequences are listed in Table 2.

#### 2.6.6. Validation of Transcriptome-Identified Signaling Pathways by Western Blot

Liver tissues were lysed in RIPA buffer (CWBio, Taizhou, China) supplemented with protease and phosphatase inhibitors to extract total protein. Protein concentrations were measured using a BCA assay kit (Beyotime, Shanghai, China). Each protein sample (25 μg) was mixed with loading buffer and denatured at 100 °C for 5 min, then separated by SDS-PAGE and transferred onto Polyvinylidene fluoride (PVDF) membranes (MilliporeSigma, Burlington, MA, USA). Membranes were blocked with 5% non-fat milk at room temperature for 1 h and then incubated overnight at 4 °C with the following primary antibodies: phosphorylated-PI3K (p-PI3K, Cat. No. AF3242, Affinity, Shanghai, China), PI3K (Cat. No. AF6241, Affinity, China), phosphorylated-AKT (p-AKT, Cat. No. AF0016, Affinity, China), AKT (Cat. No. HA721870, HuaAn, Shanghai, China), phosphorylated-NF-κB (p-NF-κB, Cat. No. AF2006, Affinity, China), NF-κB (Cat. No. AF5006, Affinity, China), and β-Actin (Cat. No. AF7018, Affinity, China) as the internal control. After washing, membranes were incubated with HRP-conjugated Goat anti-Rabbit IgG (polyclonal, Cat. No. HA1001, HuaAn, China) at room temperature for 1 h. Protein bands were visualized using a G:Box Chemi XRQ imaging system (Syngene, Cambridge, UK) and quantified by densitometry using ImageJ software (version 1.54g; NIH, Bethesda, MD, USA).

### 2.7. Statistical Analysis

#### 2.7.1. Data Statistics and Significance Testing

All statistical analyses were conducted using GraphPad Prism 10.0 (GraphPad Software, San Diego, CA, USA). Differences between groups in liver immune markers and DEG counts were evaluated using independent samples *t*-tests. Before conducting these parametric tests, we confirmed the normality of the data distribution using the Shapiro–Wilk test [24]. Data are presented as mean ± standard error of the mean (SEM), and differences were considered statistically significant at *p*-value < 0.05.

#### 2.7.2. Gene Set Enrichment Analysis (GSEA)

GSEA was performed to systematically evaluate expression trends across the entire transcriptome. All genes were ranked by log_2_ fold change values and subjected to functional enrichment analysis using the KEGG and GO databases. The analysis was performed using the cluster Profiler R package (v4.0.5), identifying signaling pathways exhibiting consistent up or downregulation trends, with significance thresholds set at FDR < 0.25 and *p*-value < 0.05 [25].

#### 2.7.3. Weighted Gene Co-Expression Network Analysis

To identify gene modules closely associated with changes in liver immune function, the WGCNA algorithm in R was applied to the transcriptome data [26]. A correlation network of gene expression across samples was constructed to detect co-expression modules exhibiting high correlations with key immune phenotypes. Co-expression networks of genes within the key modules were subsequently visualized using Cytoscape software (v3.7.1, Bethesda, MD, USA) [27]. Core regulatory factors within the network were identified using the MCODE plugin (v2.0) in Cytoscape, which clusters genes within the modules. Hub genes were defined based on the following parameters: degree cutoff = 2, K-core = 2, and maximum depth = 100 [28].

## 3. Results

### 3.1. Effects of Compound Yeast Culture on the Histomorphological Structure of Lamb Liver Tissue

**Figure 1:**
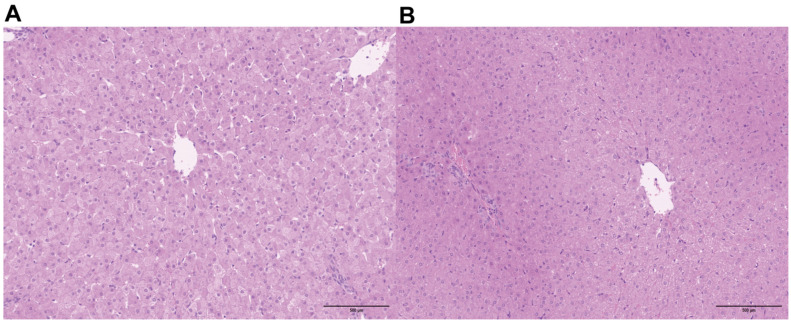
Effects of compound yeast culture (CYC) on liver histomorphology in lambs. Representative hematoxylin–eosin (H&E) stained liver sections from the control group (**A**) and the CYC-supplemented group (**B**). (**A**) The control group shows normal hepatic architecture with clearly defined hepatocytes and well-organized hepatic cords. (**B**) The CYC-supplemented group exhibits similarly normal morphology, without signs of inflammation, steatosis, or necrosis, compared with the control. All images were taken at 100× magnification. Scale bar = 500 μm.

To evaluate the effects of CYC on the histological structure of lamb liver, paraffin sections were prepared from liver tissue blocks fixed in 4% paraformaldehyde and subsequently stained with H&E. In the control group (Figure 1A), hepatic lobule architecture remained largely intact, displaying normal central vein morphology and well-aligned hepatocytes. However, certain regions exhibited widened interlobular spaces and mild sinusoidal dilation. In contrast, the CYC group (Figure 1B) displayed a more compact hepatic structure, characterized by regular interlobular arrangement and well-defined sinusoidal contours. These results suggest that CYC plays a role in ameliorating hepatic tissue pathology and maintaining a more regular architecture.

### 3.2. Effects of Compound Yeast Culture on Immune Factors in Lamb Liver Tissue

**Figure 2:**
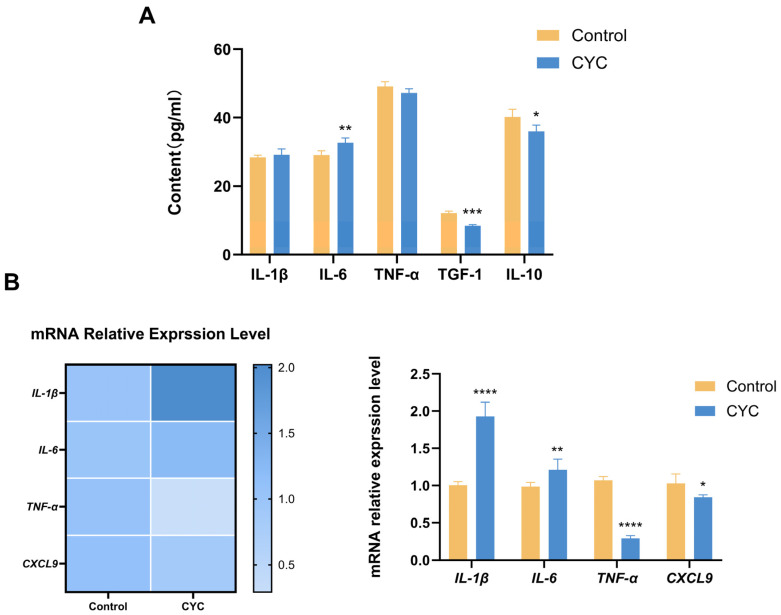
Effects of dietary CYC supplementation on hepatic cytokine expression in lambs. (**A**) Protein concentrations (pg/mL) of IL-1β, IL-6, TNF-α, TGF-β1, and IL-10 in liver tissue, measured by ELISA. (**B**) Relative mRNA expression levels of *IL-1β*, *IL-6*, *TNF-α*, and *CXCL9* in liver tissue, quantified by qRT-PCR. Data are displayed as a heatmap and a corresponding bar chart. Values are presented as mean ± SEM. * *p*-value < 0.05, ** *p*-value < 0.01, *** *p*-value < 0.001, **** *p*-value < 0.0001.

To further evaluate the impact of CYC on hepatic immune status, IL-1β, IL-6, IL-10, TNF-α, TGF-β1, and CXCL9 levels were assessed in liver tissue using ELISA and qRT-PCR. Compared with the control group, IL-6 protein levels were notably elevated (*p*-value < 0.01) in the CYC group, whereas IL-10 (*p*-value < 0.05) and TGF-β1 (*p*-value < 0.0001) were substantially reduced. No obvious differences were observed in IL-1β or TNF-α protein abundance. At the mRNA level, qRT-PCR results showed *IL-1β* (*p*-value < 0.0001) and *IL-6* (*p*-value < 0.01) expression was strikingly upregulated, while *TNF-α* (*p*-value < 0.001) and *CXCL9* (*p*-value < 0.05) were distinctly downregulated. These results indicate that CYC modulates the hepatic immune environment, characterized by a shift in cytokine expression and production (Figure 2).

### 3.3. Effects of Compound Yeast Culture on Hepatic Antioxidant and Oxidative Stress Parameters in Lambs

**Figure 3:**
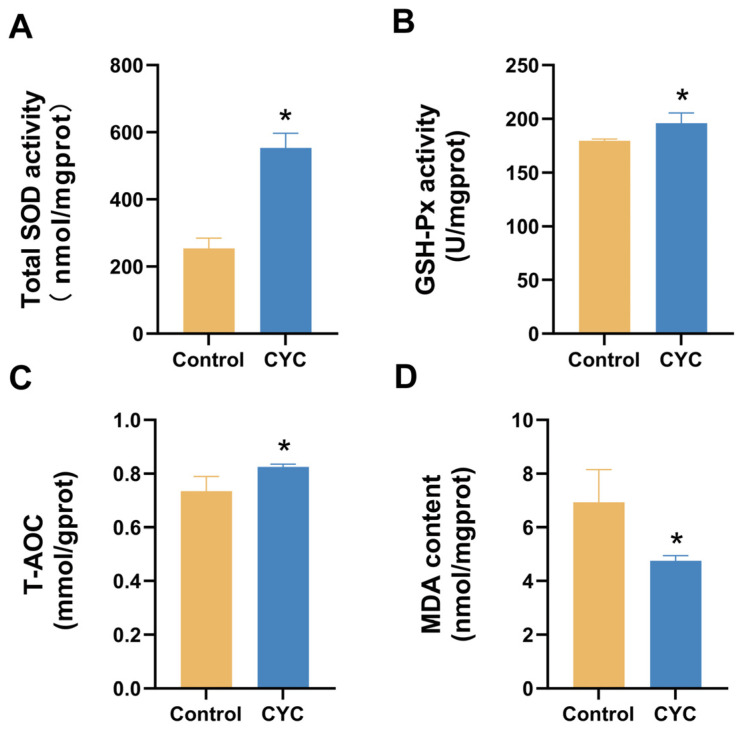
Effects of compound yeast culture (CYC) on hepatic oxidative stress parameters in lambs. (**A**) T-SOD, (**B**) GSH-Px, (**C**) T-AOC, and (**D**) MDA were measured in liver tissue. Data are expressed as mean ± SEM. * *p*-value < 0.05 compared with the control group.

To evaluate the effect of CYC on hepatic oxidative stress, the activities of T-SOD, GSH-Px, T-AOC, and the content of MDA were measured (Figure 3). Compared with the control group, T-SOD, GSH-Px, and T-AOC activities (Figure 3A–C) were all significantly increased in the CYC group, while MDA content (Figure 3D) was significantly decreased, demonstrating that CYC supplementation simultaneously enhances multiple antioxidant enzymes and reduces lipid peroxidation in the liver.

### 3.4. Transcriptomic Analysis of Lamb Liver Following Compound Yeast Culture Supplementation

Transcriptome sequencing of ten lamb liver samples identified a total of 30,724 genes. A total of 98.81 GB of high-quality clean data was generated, with each sample contributing over 5.92 GB. The Q20 and Q30 read coverage rates exceeded 98.53% and 95.42%, respectively, satisfying standard sequencing quality thresholds (Table 3). The sequencing error rate was below 0.0123%, further confirming the high quality of the transcriptome data. Overall, the sequencing metrics confirmed that the data met the criteria for transcriptome analysis and were suitable for downstream analyses.

**Figure 4:**
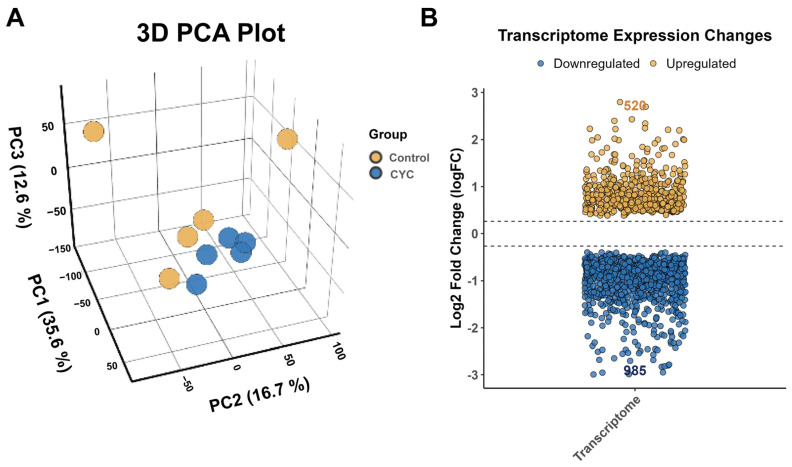
Transcriptome expression analysis and identification of differentially expressed genes (DEGs) in lamb liver. (**A**) Three-dimensional principal component analysis (3D PCA) plot based on the expression profiles of all detected genes. Each sphere represents an individual sample, colored by group: control (yellow) and CYC (blue). The axes correspond to the first three principal components (PCs), with the percentage of total variance explained by each shown in parentheses. (**B**) Volcano plot showing the distribution of DEGs between groups. Compared with the control group, 520 genes were significantly upregulated (yellow dots) and 985 genes were significantly downregulated (blue dots) in the CYC group. DEGs were identified using the DESeq2 package with thresholds of |log_2_ Fold Change| > 1 and FDR < 0.05.

Three-dimensional principal component analysis (3D PCA) revealed clear separation between the control and CYC groups, indicating distinct transcriptional profiles in the liver. Differential expression analysis was performed using the DESeq2 package, with thresholds of |log_2_ Fold Change| > 1 and false discovery rate (FDR) < 0.05. A total of 1505 DEGs were identified, including 520 upregulated and 985 downregulated genes in the CYC group compared to the control (Figure 4).

#### 3.4.1. Gene Ontology (GO) Functional Analysis of DEGs

**Figure 5:**
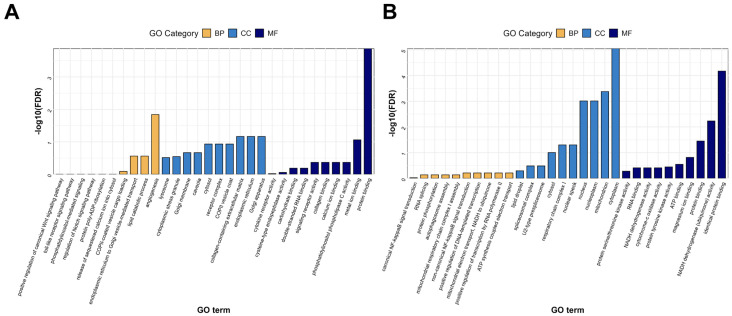
Gene Ontology (GO) enrichment analysis of differentially expressed genes (DEGs). Bar charts show the top significantly enriched GO terms for (**A**) upregulated DEGs and (**B**) downregulated DEGs in lamb liver. The x-axis indicates the GO terms, and the y-axis represents statistical significance as –log_10_ (FDR). Bars are color-coded according to the three main GO categories: Biological Process (BP, yellow), Cellular Component (CC, light blue), and Molecular Function (MF, dark blue). GO enrichment analysis was performed using a significance threshold of FDR < 0.05.

To further elucidate the regulatory mechanisms of CYC on gene expression in sheep liver, GO enrichment analysis was conducted separately for upregulated and downregulated DEGs. Upregulated genes were significantly enriched in 66 GO terms (FDR < 0.05), primarily involved in lipid metabolism and other biological (Table S2). Cellular component enrichment included the cytoplasm and Golgi apparatus, while molecular functions were associated with protein binding and phosphatase activity (Figure 5A). In contrast, downregulated genes were enriched in 75 GO terms (FDR < 0.05), mainly associated with cellular components including the nucleus and mitochondria (Table S3). Their molecular functions comprised NADH dehydrogenase activity and protein binding, and biological processes were linked to inflammatory responses, including the NF-κB signaling pathway (Figure 5B). Overall, these results indicate that CYC intervention may enhance liver function and homeostasis by upregulating metabolism and protein processing-related pathways while concurrently suppressing inflammation and oxidative stress-associated pathways.

#### 3.4.2. KEGG Enrichment Analysis of Functional Pathways for DEGs

**Figure 6:**
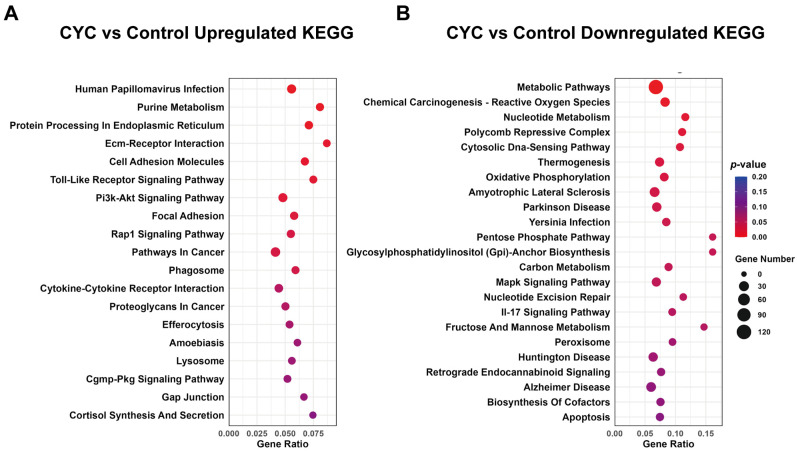
Kyoto Encyclopedia of Genes and Genomes (KEGG) enrichment analysis of differentially expressed genes (DEGs) in lamb liver. (**A**) KEGG pathways enriched by upregulated DEGs in the CYC group compared to the Control group. (**B**) KEGG pathways enriched by downregulated DEGs. The x-axis represents enrichment scores, while the y-axis indicates the corresponding pathways. Bubble size reflects the number of genes in each pathway, and bubble color represents the significance level.

To further explore the functional pathways associated with the DEGs, KEGG enrichment analysis was conducted. Upregulated DEGs were notably enriched in pathways related to metabolic regulation, signal transduction, and protein processing. Genes enriched in the purine metabolism pathway (*GUCY2C*, *ADCY1*, *PDE5A*), involved in cyclic guanosine monophosphate synthesis and degradation, suggest that CYC may modulate energy metabolism and cellular signaling efficiency via second messenger regulation [29]. Upregulated genes in the PI3K-AKT signaling pathway (*ITGB1*, *ANGPT1*, *LAMC1*), involved in cell adhesion, proliferation, and survival, may contribute to the homeostasis and repair in hepatic tissue. Increased expression of key receptors and transducers in the Toll-like receptor pathway (*TLR2*, *TLR8*, *IRAK4*) indicates that CYC may enhance innate immune recognition and responses to microbe-associated molecular patterns [30]. Additionally, significant enrichment in the endoplasmic reticulum protein processing pathway (*ERO1A*, *SAR1A*) suggests enhanced protein folding and transport capacity, potentially as an adaptive response during immune activation (Table S4). Conversely, downregulated DEGs were predominantly enriched in pathways associated with basal metabolism, oxidative stress, and disease. For example, downregulation of genes including *COMT*, *DGAT1*, and *PSPH* may suppress energy-intensive metabolic processes, reducing hepatocyte metabolic load. Suppressed expression of genes in ROS-related chemotransformation (*MAP2K2*, *VEGFA*, *MAPK13*) and MAPK signaling pathways (*NFKB2*, *RELB*, *ELK1*) suggests that CYC may mitigate inflammation-induced oxidative stress and cellular damage by downregulating stress-related pathways. KEGG analysis revealed that upregulated DEGs (Figure 6A) induced by CYC were mainly associated with immune regulation and cellular structure repair, whereas downregulated DEGs (Figure 6B) were enriched in metabolic and stress-related pathways (Table S5). These findings suggest that CYC plays a multifaceted role in regulating hepatic biological functions.

#### 3.4.3. Gene Set Enrichment Analysis Verification of Systematic Enrichment Trends in Key Pathways

**Figure 7:**
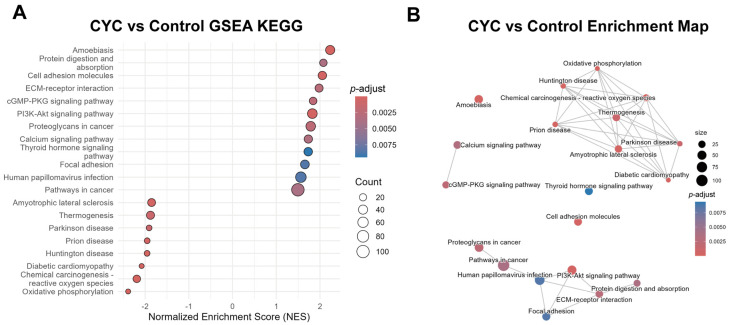
Gene Set Enrichment Analysis (GSEA) of KEGG pathways in lamb liver. (**A**) Bubble plot of enriched KEGG pathways identified by GSEA. The x-axis represents the Normalized Enrichment Score (NES). A positive NES indicates pathway enrichment in the CYC supplemented group (e.g., PI3K-AKT signaling pathway, ECM–receptor interaction), while a negative NES indicates enrichment in the control group (e.g., Oxidative phosphorylation). The y-axis lists the pathway names. The bubble size corresponds to the Count (the number of genes within that pathway’s gene set). The bubble color indicates the *p*-adjust, with red representing higher statistical significance. (**B**) Enrichment map visualizing the functional relationships between the significant pathways. Each node represents one KEGG pathway. The size of the node represents the count of enriched genes.

To systematically assess the expression trends of DEGs in enriched pathways, we performed GSEA using the complete gene expression matrix. This method enables a comprehensive evaluation of gene set enrichment patterns and potential biological functions across treatment groups. GSEA results indicated that, relative to the control group, pathways related to cellular structure, signaling, and immune regulation were distinctly upregulated in the CYC group. Notably, these included the Cell Adhesion Molecules pathway, ECM–Receptor Interaction pathway, PI3K-AKT signaling pathway, and cGMP-PKG signaling pathway (normalized enrichment score > 1, *p*-adjust < 0.05). The overall enrichment of these pathways suggests that CYC supplementation may enhance the liver’s regulatory capacity in terms of structural stability, intercellular communication, and immune responses (Figure 7A).

Network association analysis of pathway enrichment revealed distinct functional correlations and clustering among enriched pathways. Modules centered on PI3K-related pathways and ECM-related pathways (e.g., Focal adhesions and ECM–receptor interactions) formed highly interconnected clusters. Their close association suggests that these pathways may exert synergistic regulatory effects across CYC groups. Notably, PI3K and ECM-related pathways contained prominent nodes within their respective modules, whereas oxidative phosphorylation nodes were the largest and centrally positioned within the metabolic module. Together, these three pathways served as key regulatory nodes in both treatment groups (Figure 7B).

#### 3.4.4. WGCNA of Host Transcriptome Correlation with Hepatic Inflammatory Factors

**Figure 8:**
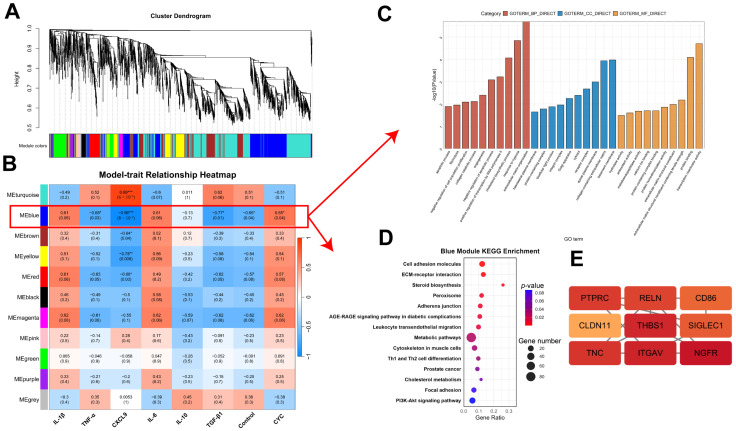
Weighted Gene Co-Expression Network Analysis Reveals Functional Modules and Hub Genes Associated with Immune Regulation in Lamb Liver. (**A**) Hierarchical clustering dendrogram of all expressed genes. Each leaf represents a gene, and the colored bar below the dendrogram (Module colors) indicates the identified co-expression modules. (**B**) Module-trait relationship heatmap. Each row corresponds to a module eigengene (ME), and each column corresponds to a trait (immune markers or experimental group). In each cell, the top number represents the Pearson correlation coefficient (r), and the bottom number in parentheses is the corresponding *p*-value. The color scale on the right indicates correlation from strongly positive (red, r = 1) to strongly negative (blue, r = −1). (**C**) GO enrichment analysis for the MEblue module. The y-axis represents statistical significance as −log10 (*p*-value). Bars are color-coded by GO category: Biological Process (red), Cellular Component (blue), and Molecular Function (orange). (**D**) KEGG pathway enrichment analysis for the MEblue module. The x-axis (Gene Ratio) represents the proportion of module genes annotated to each pathway. Bubble size indicates the gene count, and bubble color represents the *p*-value, with red indicating higher significance. (**E**) Network visualization of the top hub genes within the MEblue module. Nodes represent genes, and the color gradient (red to orange) reflects high connectivity (kME) within the module. * *p*-value < 0.05, ** *p*-value < 0.01, *** *p*-value < 0.001.

WGCNA was performed to explore transcriptional modules associated with hepatic immune responses by integrating liver transcriptome data with key cytokines (*IL-1β*, *TNF-α*, *CXCL9*, IL-6, IL-10, and TGF-β1). The MEblue module, containing 767 genes, showed strong positive correlations with IL-1β and IL-6 and a negative correlation with TGF-β1, suggesting its central role in promoting pro-inflammatory activity while suppressing anti-inflammatory signaling (Figure 8A,B). Functional enrichment analysis indicated that MEblue genes were mainly involved in immune regulation and structural pathways, including cell adhesion molecules, ECM–receptor interactions, T cell differentiation, and the PI3K-AKT signaling pathway (Figure 8C,D). Hub gene analysis identified PTPRC, CD86, ITGAV, THBS1, and CLDN11 as key regulators of immune recognition, T-cell activation, and cell adhesion (Figure 8E). Together, these results highlight the MEblue module as a critical transcriptional network mediating the hepatic immune-modulatory effects of CYC (Tables S6 and S7).

### 3.5. qRT-PCR Validation of Transcriptome Results

**Figure 9:**
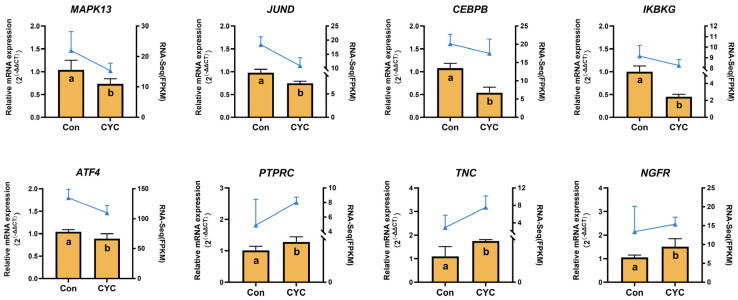
qRT-PCR validation of RNA sequencing (RNA-Seq) results for *MAPK13*, *JUND*, *CEBPB*, *IKBKG*, *ATF4*, *PTPRC*, *TNC*, and *NGFR* in lamb liver tissue. Data are presented as mean ± SEM; a, b indicate statistically significant differences between groups/treatments at *p*-value < 0.05.

qRT-PCR was performed to validate the transcriptome sequencing results and to confirm the differential expression of eight genes identified by WGCNA as closely associated with the PI3K-AKT and CAMs signaling pathways: MAPK13, JUND, CEBPβ, IKBKγ, ATF4, PTPRC, TNC, and NGFR. The expression patterns detected by qRT-PCR were consistent with RNA-Seq results (Figure 9). Specifically, CYC supplementation significantly increased the expression of PTPRC, TNC, and NGFR (*p*-value < 0.05) in lamb liver, whereas MAPK13, JUND, CEBPβ, IKBKγ, and ATF4 were significantly downregulated. These results confirm the reliability of the transcriptomic data and indicate that these genes may play pivotal roles in mediating CYC-induced regulation of the PI3K-AKT signaling pathway and cell adhesion processes.

### 3.6. Western Blot Validation of PI3K-AKT and NF-κB Pathway Activation

**Figure 10:**
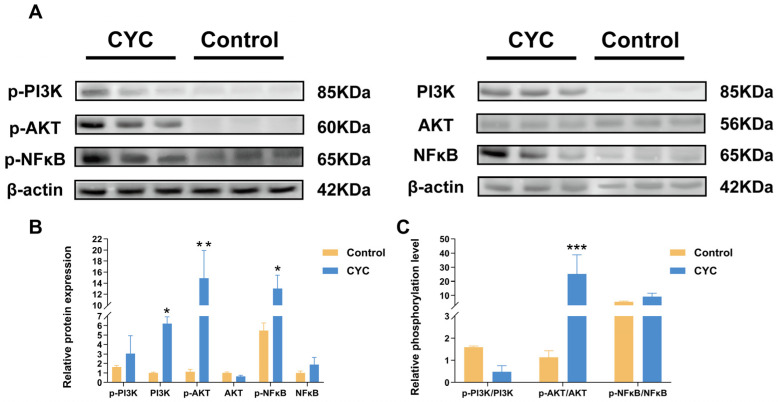
Effects of compound yeast culture (CYC) on PI3K, AKT and NF-κB signaling pathway in lamb liver. (**A**) Immunoblot analysis of total and phosphorylated forms of PI3K, AKT, and NF-κB in liver tissues. (**B**) Quantification of total and phosphorylated protein expression normalized to β-actin. (**C**) Relative phosphorylation levels of PI3K, AKT, and NF-κB (ratio of phosphorylated to total protein, phosphorylated/total protein ratio). Data are presented as mean ± SEM. * *p*-value < 0.05, ** *p*-value < 0.01, *** *p*-value < 0.001 compared with the Control group.

Western blot analysis was used to evaluate the effects of CYC on hepatic PI3K-AKT and NF-κB signaling (Figure 10A). Quantification of protein expression (Figure 10B) showed that total PI3K (*p*-value < 0.05), p-AKT (*p*-value < 0.01) and p-NF-κB (*p*-value < 0.05) were significantly upregulated in the CYC group. However, no significant differences were observed in the expression of p-PI3K, total AKT, or total NF-κB proteins. Further analysis of the phosphorylation ratio (Figure 10C) revealed a robust increase in AKT activation (p-AKT/AKT) in the CYC group (*p*-value < 0.001). In contrast, the phosphorylation ratios of PI3K and NF-κB remained unchanged. These findings indicate that CYC treatment significantly enhances AKT activation and increases PI3K protein expression, while the phosphorylation ratio of NF-κB remains unchanged.

## 4. Discussion

In ruminants, the liver functions as a central hub for metabolism and immune regulation, critically responding to exogenous stressors [31] and pathogen invasion. The integrity of hepatic tissue and the homeostasis of immune factors are closely linked to overall animal health [32,33]. In our study, histological analysis revealed that CYC markedly enhanced hepatocyte alignment, improved the regularity of hepatic cords, and maintained the continuity of sinusoidal structures, indicating that CYC exerts significant hepatoprotective effects. Such structural improvements likely stabilize the hepatic microenvironment, providing a basis for subsequent regulation of immune and metabolic functions [34].

Inflammatory cytokines are pivotal in maintaining immune homeostasis, and their expression profiles reflect the host’s response to immunomodulatory interventions [35]. Canonical pro-inflammatory cytokines, including IL-1β and IL-6, are rapidly secreted by innate immune cells, such as macrophages and dendritic cells, in response to pathogen detection or tissue injury. These cytokines primarily initiate immune responses, trigger acute-phase reactions, and promote chemotaxis [36]. Previous studies have shown that yeast and its derivatives enhance IL-1β and IL-6 expression in the liver, thereby augmenting immune activity in animals [37,38]. Further research indicates that dietary yeast supplementation elevates serum levels of pro-inflammatory factors, including IL-1β, IL-6, IFN-γ, and TNF-α in lambs [39]. The main active component of yeast cultures, β-glucan, functions as a cellular response modulator, directly engaging pattern recognition receptors (PRRs) like TLRs and Dectin-1. β-Glucan modulates the immune system, influencing both innate and adaptive immunity by stimulating immune cells and inducing cytokine production [39].

Specific components in CYC, including β-glucan and mannan, modulate inflammatory cytokine expression by binding to PRRs on macrophages and neutrophils [10]. In this study, CYC treatment significantly upregulated the expression of pro-inflammatory factors IL-1β and IL-6, while TNF-α, CXCL9, TGF-β1, and IL-10 were downregulated at the transcriptional level. Notably, although TNF-α is a classical pro-inflammatory cytokine, sustained or excessive expression in the liver is often linked to apoptosis, tissue necrosis, and inflammatory injury. Therefore, its downregulation may reflect a protective mechanism that limits tissue damage [40]. CXCL9, a key ligand of the CXCR3 axis, has been shown in animal models to recruit effector T cells and exacerbate tissue inflammation. Consequently, CXCL9 downregulation may limit excessive immune cell recruitment and reduce local tissue damage [41]. TGF-β1 and IL-10 are well-established regulatory cytokines involved in immunosuppression and immune tolerance. High levels of these cytokines are frequently linked to immunosuppression, chronic inflammation, and fibrosis. Therefore, moderate reductions in TGF-β1 and IL-10 during weaning stress may prevent premature immune suppression and preserve effective early immune responses [42,43]. In summary, the observed upregulation of IL-1β and IL-6, coupled with downregulation of TNF-α, CXCL9, TGF-β1, and IL-10, indicates bidirectional immune regulation. This mechanism rapidly activates immune responses through early pro-inflammatory factors while simultaneously restraining pathways that could lead to tissue damage or excessive immune cell recruitment. Overall, this regulation maintains hepatic immune homeostasis during weaning stress.

KEGG enrichment analysis revealed that CYC-regulated DEGs were significantly enriched in immune-related pathways, particularly the PI3K-AKT signaling pathway, Toll-like receptor signaling, and cell adhesion molecules. The PI3K-AKT pathway functions as a central hub linking inflammatory responses to tissue homeostasis, regulating immune cell migration and tissue repair [44,45]. Its key effector, AKT, is activated in a PI3K-dependent manner, which subsequently triggers NF-κB signaling and induces pro-inflammatory cytokines such as TNF-α and IL-6 [46]. This mechanistic framework is consistent with the observed upregulation of IL-6 and IL-1β in the CYC group. Moreover, β-glucan, a major yeast cell wall component, can interact with TLRs (e.g., TLR2) through the Dectin-1 receptor, thereby stimulating IL-6 and TNF-α production and enhancing innate immune responses [47]. The observed upregulation of TLR2 and TLR8 further supports that CYC may enhance hepatic recognition of pathogen-associated molecular patterns (PAMPs). Western blot analysis confirmed that CYC treatment markedly increased PI3K expression and AKT phosphorylation, reflecting enhanced PI3K-AKT signaling in the liver. Although p-NF-κB levels increased, the unchanged phosphorylation ratio suggests limited engagement of the canonical NF-κB pathway. These protein-level findings corroborate the transcriptomic data, together suggesting that CYC enhances hepatic immune surveillance by promoting pathogen sensing, immune cell adhesion, and migration in weaned lambs.

To validate the overall transcriptional trends beyond threshold-based DEG screening, GSEA was performed. The results confirmed significant activation of the ECM–receptor interaction, PI3K-AKT, and CAM pathways, which was consistent with KEGG findings. These coordinated changes suggest synergistic regulation of immune function and cellular architecture. Previous studies have reported that conjugated linoleic acid maintains rumen epithelial homeostasis by modulating ECM–receptor and CAM pathways [48], further supporting the structural relevance of these findings. Complementarily, WGCNA integrated with cytokine profiles identified a key module (MEblue) significantly correlated with inflammatory markers. This module was enriched in pathways related to PI3K-AKT signaling, T cell differentiation, and extracellular matrix remodeling, further confirming the central role of these pathways in CYC-mediated hepatic immune regulation.

In parallel, KEGG analysis of downregulated DEGs revealed enrichment in oxidative stress-related pathways, including MAPK signaling and chemotaxis. Since activation of NF-κB and MAPK cascades is closely associated with ROS production and tissue damage [49], these transcriptomic results suggest that CYC may mitigate weaning stress-induced ROS accumulation. This inference was directly supported by our biochemical findings. Weaning stress is known to disrupt redox balance by reducing antioxidant defenses, such as SOD, GSH-Px, and T-AOC, while increasing lipid peroxidation, as indicated by elevated MDA levels [50]. In the present study, CYC supplementation significantly enhanced hepatic T-AOC and bolstered the activities of key antioxidant enzymes, including total SOD and GSH-Px. This coordinated enhancement of the endogenous antioxidant defense system effectively counteracted lipid peroxidation, as evidenced by the significantly reduced MDA content in the CYC group. These results are consistent with previous reports showing that yeast supplementation improves antioxidant status in ruminants under stress conditions [12,51]. Collectively, the data imply that CYC affects hepatic immune function and antioxidant defenses, which could help preserve liver homeostasis under weaning stress.

Collectively, our findings demonstrate that CYC regulates hepatic immune responses through activation of the TLR and PI3K-AKT pathways, thereby enhancing immune recognition and cell–cell interactions. At the same time, CYC suppresses excessive MAPK signaling and oxidative stress responses, alleviating cellular injury. This dual action contributes to maintaining hepatic homeostasis in weaned lambs, providing mechanistic evidence for the application of CYC as a functional microecological feed additive.

This study is limited by the small number of animals and the focus on in vivo experiments, which precluded direct assessment of CYC’s effects on isolated hepatic cells. Hepatic homeostasis involves multiple factors, and only a subset of pathways was examined. Further studies with larger cohorts and complementary in vitro approaches are needed to validate these findings and clarify the underlying mechanisms.

## 5. Conclusions

**Figure 11:**
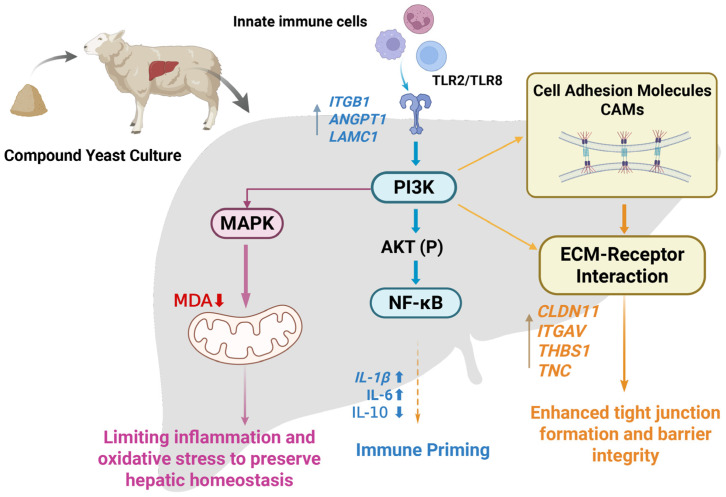
Schematic representation of the proposed mechanisms by which CYC regulates hepatic immune function and homeostasis in weaned lambs. CYC supplementation activates the PI3K–AKT signaling axis, serving as a central hub for: (1) Immune Priming—modulating NF-κB to uregulate *IL-1β* and IL-6 while downregulating IL-10, enhancing immune surveillance; (2) Oxidative Stability—reducing lipid peroxidation (MDA); (3) Barrier Integrity—promoting tight junction formation through CAMs and ECM–receptor genes (e.g., *ITGB1*, *CLDN11*). Solid arrows indicate direct regulation; dashed arrows indicate modulated or downstream effects.

This study demonstrates that dietary supplementation with compound yeast culture (CYC) enhances hepatic immune regulation in weaned lambs (Figure 11, created with BioRender; https://www.biorender.com, accessed on 1 December 2025). CYC improved liver morphology, modulated inflammatory cytokine expression, and activated PI3K-AKT signaling, as evidenced by upregulated PI3K and robust AKT phosphorylation. Although p-NF-κB levels were elevated, the unchanged phosphorylation ratio suggests limited engagement of the canonical NF-κB pathway. In parallel, antioxidant enzyme activities were elevated and lipid peroxidation reduced, indicating improved hepatic oxidative status. CYC also promoted tight junction formation and barrier integrity, supporting structural and functional maintenance of the liver. Together, these coordinated effects suggest that CYC reinforces hepatic immune responsiveness, preserves barrier function, and enhances oxidative stability, establishing it as a promising nutritional strategy for promoting liver health and stress resilience in ruminants.

## Figures and Tables

**Table 1:** Primer Sequences for qRT-PCR.

Gene Sequence Number	Gene Name	Primer Sequence (5′–3′)	Fragment Size
NM_001009465.2	*IL-1β*	F: TGCTGGATAGCCCATGTGTG	84 bp
		R: CGAAGCTCATGCAGAACACC	
NM_001009392.1	*IL-6*	F: TCATGGAGTTGCAGAGCAGT	137 bp
		R: TGCGTTCTTTACCCACTCGT	
NM_001024860.1	*TNF-α*	F: ATAACAAGCCGGTAGCCCAC	82 bp
		R: AGGGCATTCGCATACGAGTC	
XM_004009924.5	*CXCL9*	F: GAGTTCAAGGAATCCCAGCAAT	121 bp
		R: TCACAAGTAGGGCTTGGAGC	
NM_001190390.1	*GAPDH*	F: CGGCACAGTCAAGGCAGAGAAC	115 bp
		R: CACGTACTCAGCACCAGCATCAC	

**Table 2:** Primer Sequences for qRT-PCR.

Gene Sequence Number	Gene Name	Primer Sequence (5′–3′)	Fragment Size
NM_001139455.1	*MAPK13*	F: TCACCCGGAAAAAGGGCTTC	88 bp
		R: CCTATGTGCGTCAGGGACAC	
XM_027969415.2	*JUND*	F: GCTCAAGGATGAACCGCAGA	98 bp
		R: CCTTAATGCGCTCTTGCGTG	
XM_004014883.5	*CEBPB*	F: CCCGCCCGTGGTGTTATTT	124 bp
		R: ATCAACTTCGAAACCGGCCC	
XM_060408446.1	*IKBKG*	F: CTCACCCAAGGGAGGAGTGA	80 bp
		R: GATCGCCCTGTCGTACATCC	
XM_060411578.1	*ATF4*	F: GGACGGCCATCGATTTTGTG	113 bp
		R: GATCGCCCTGTCGTACATCC	
XM_042229308.1	*PTPRC*	F: GGTCCTTCCACTCAAGACACCT	89 bp
		R: GCTGTTGTGGTGAGACTGTGTG	
XM_027974687.2	*NGFR*	F: TAGCATGAACAAGCCCCGAG	122 bp
		R: TCAGGTCAAAGAAGTGCGGT	
XM_042242806.2	*TNC*	F: CAGGAACCCAGAGGAAGCTG	82 bp
		R: CCTTGGGTGAAGCCAGAGAC	
NM_001190390.1	*GAPDH*	F: CGGCACAGTCAAGGCAGAGAAC	115 bp
		R: CACGTACTCAGCACCAGCATCAC	

**Table 3:** Transcriptome Sequencing Data Quality Summary.

Sample	Raw Reads	Clean Reads	Clean Bases	Error Rate (%)	Phred > 20 Q20 (%)	Phred > 30 Q30 (%)	GC Content (%)
Con1	40,795,574	40,415,582	6,007,157,449	0.0122	98.61	95.71	42.55
Con2	45,443,212	44,989,736	6,706,481,259	0.0123	98.53	95.42	48.49
Con3	41,117,388	40,759,796	6,064,085,751	0.0121	98.66	95.88	42.43
Con4	44,108,830	43,730,030	6,501,297,594	0.0121	98.63	95.77	45.85
Con5	51,050,188	50,569,256	7,532,453,630	0.0122	98.58	95.60	49.09
CYC1	47,973,624	47,536,592	7,094,922,014	0.0122	98.58	95.61	47.13
CYC2	46,513,486	46,084,096	6,859,413,840	0.0122	98.6	95.65	47.44
CYC3	45,482,228	45,067,310	6,725,812,139	0.0122	98.59	95.62	47.40
CYC4	43,768,544	43,324,786	6,463,150,497	0.0123	98.57	95.57	48.42
CYC5	50,995,150	50,530,560	7,547,720,177	0.0122	98.61	95.69	47.33

## Data Availability

The data presented in this study are openly available in Sequence Read Archive at https://submit.ncbi.nlm.nih.gov/subs/sra/ (accessed on 20 October 2025), reference number PRJNA1332615.

## Supplementary Materials

The following supporting information can be downloaded at: https://www.mdpi.com/article/10.3390/ani16010104/s1. Table S1: Nutrient composition of mixed feed and the main active compound yeast culture (feed-based); Table S2: GO enrichment analysis results of DEGs upregulated in liver tissue; Table S3: GO enrichment analysis results of DEGs downregulated in liver tissue; Table S4: KEGG enrichment analysis results of DEGs upregulated in liver tissue; Table S5: KEGG enrichment analysis results of DEGs downregulated in liver tissue; Table S6. GO enrichment results of the MEblue in WGCNA results; Table S7: KEGG enrichment results of the MEblue in WGCNA results; Figure S1: Original uncropped Western blot membrane for p-PI3K, corresponding to the assembled blot shown in Figure 10; Figure S2: Original uncropped Western blot membrane for PI3K, corresponding to the assembled blot shown in Figure 10; Figure S3: Original uncropped Western blot membrane for p-AKT, corresponding to the assembled blot shown in Figure 10; Figure S4: Original uncropped Western blot membrane for AKT, corresponding to the assembled blot shown in Figure 10; Figure S5: Original uncropped Western blot membrane for p-NF-κB, corresponding to the assembled blot shown in Figure 10; Figure S6: Original uncropped Western blot membrane for NF-κB, corresponding to the assembled blot shown in Figure 10; Figure S7: Original uncropped membrane showing the β-actin internal control used for normalization of the phosphorylated proteins in Figure 10; Figure S8: Original uncropped membrane showing the β-actin internal control used for normalization of the total proteins in Figure 10.

## Abbreviations

The following abbreviations are used in this manuscript:

CYC	compound yeast culture
WGCNA	Weighted Gene Co-expression Network Analysis
GSEA	Gene Set Enrichment Analysis
PI3K-AKT	Phosphatidylinositol 3-kinase–AKT
ECM–receptor	Extracellular matrix–receptor
TLRs	Toll-like receptors
MAPK	Mitogen-activated protein kinase
ROS	Reactive oxygen species
IL-1β	Interleukin-1 beta
IL-6	Interleukin-6
IL-10	Interleukin-10
TNF-α	Tumour necrosis factor-alpha
TGF-β1	Transforming growth factor-beta 1
CXCL9	C-X-C motif chemokine ligand 9
PTPRC	Protein Tyrosine Phosphatase Receptor Type C (Hub Gene)
CD86	Cluster of Differentiation 86 (Hub Gene)
ITGAV	Integrin Subunit Alpha V (Hub Gene)
CAMs	Cell Adhesion Molecules
TMR	Total mixed ration
PBS	Phosphate-buffered saline
qRT-PCR	quantitative reverse transcription (qRT)-PCR
H&E	Haematoxylin and eosin
ELISA	Enzyme-linked immunosorbent assay
DEGs	Differentially expressed genes
TPM	Transcripts per million
FDR	False discovery rate
GO	Gene Ontology
KEGG	Kyoto Encyclopedia of Genes and Genomes
3D PCA	Three-dimensional principal component analysis
cGMP-PKG	Cyclic guanosine monophosphate–protein kinase G
NF-κB	Nuclear factor kappa B
PRRs	Pattern recognition receptors
PAMPs	Pathogen-associated molecular patterns
SEM	Standard Error of the Mean
T-SOD	Total superoxide dismutase
GSH-Px	Glutathione peroxidase
T-AOC	Total antioxidant capacity
MDA	Malondialdehyde
