# Supplementary material for: Immune-Modulatory Mechanism of Compound Yeast Culture in the Liver of Weaned Lambs
Source: Animals (Basel). 2025 Dec 30;16(1):104. doi: 10.3390/ani16010104 (PMC12784882; doi:10.3390/ani16010104)
Supplement: Supplementary file 1 [file animals-16-00104-s001.zip › animals-3969586-supplementary.pdf]

Immune-Modulatory Mechanism of compound yeast culture in the Liver of Weaned Lambs

Chenlu Li[1†], Hui Bai[1,2†], Pengxiang Bai[1], Chenxue Zhang[1], Yuan Wang[1], Dacheng Liu[1,2*], Hui Chen[1,2*]

Table S1. Nutrient composition of mixed feed and The main active compound yeast culture (feed-based)

Item	Mixed feed	Compound yeast culture
Crude protein, %	19.23	20.39
Dry matter, %	92.04	93.21
Neutral detergent fiber, %	34.11	34.23
Acid detergent fiber, %	20.02	19.97
Live yeast cells, CFU/g		7.2×10^6
Lactic acid, mmol/kg		380.31

Table S2. GO enrichment analysis results of DEGs up-regulated in liver tissue

GO Term	Count	*p*-value	Gene Ratio%
GO:0005515 : protein binding	90	0.000000223	17.64705882
GO:0001525 : angiogenesis	11	0.000012100	2.156862745
GO:0062023 : collagen-containing extracellular matrix	12	0.000286000	2.352941176
GO:0046872 : metal ion binding	65	0.000292000	12.74509804
GO:0005783 : endoplasmic reticulum	26	0.000604000	5.098039216
GO:0006888 : endoplasmic reticulum to Golgi vesicle-mediated transport	10	0.000652000	1.960784314
GO:0016042 : lipid catabolic process	8	0.000690000	1.568627451
GO:0005794 : Golgi apparatus	23	0.000731000	4.509803922

GO:0005829 : cytosol	60	0.001721895	11.76470588
GO:0043235 : receptor complex	10	0.002198589	1.960784314
GO:0030127 : COPII vesicle coat	4	0.002492627	0.784313725
GO:0090110 : COPII-coated vesicle cargo loading	4	0.002735206	0.784313725
GO:0038023 : signaling receptor activity	9	0.003063748	1.764705882
GO:0005518 : collagen binding	5	0.00349431	0.980392157
GO:0005509 : calcium ion binding	28	0.003790246	5.490196078
GO:0004435 : phosphatidylinositol phospholipase C activity	4	0.004325413	0.784313725
GO:0090263 : positive regulation of canonical Wnt signaling pathway	6	0.005197979	1.176470588
GO:0000139 : Golgi membrane	17	0.00588873	3.333333333
GO:0005901 : caveola	5	0.006135537	0.980392157
GO:0030246 : carbohydrate binding	12	0.00765582	2.352941176
GO:0003725 : double-stranded RNA binding	6	0.008742569	1.176470588
GO:0010494 : cytoplasmic stress granule	6	0.009140376	1.176470588
GO:0002224 : toll-like receptor signaling pathway	4	0.009702791	0.784313725
GO:0005764 : lysosome	10	0.011948374	1.960784314
GO:0022626 : cytosolic ribosome	3	0.012142281	0.588235294
GO:0005789 : endoplasmic reticulum membrane	25	0.013034602	4.901960784
GO:0004197 : cysteine-type endopeptidase activity	6	0.013267971	1.176470588
GO:0048015 : phosphatidylinositol-mediated signaling	4	0.014342995	0.784313725

GO:0009897 : external side of plasma membrane	16	0.016024218	3.137254902
GO:0004896 : cytokine receptor activity	5	0.016139957	0.980392157
GO:0005654 : nucleoplasm	31	0.016162385	6.078431373
GO:0008593 : regulation of Notch signaling pathway	3	0.016377451	0.588235294
GO:0070212 : protein poly-ADP-ribosylation	3	0.016377451	0.588235294
GO:0070971 : endoplasmic reticulum exit site	4	0.016527883	0.784313725
GO:0051209 : release of sequestered calcium ion into cytosol	4	0.018022391	0.784313725
GO:0042803 : protein homodimerization activity	11	0.018087522	2.156862745
GO:0030509 : BMP signaling pathway	6	0.018445534	1.176470588
GO:0007265 : Ras protein signal transduction	5	0.019607304	0.980392157
GO:1990253 : cellular response to leucine starvation	3	0.020170116	0.588235294
GO:0034975 : protein folding in endoplasmic reticulum	3	0.020170116	0.588235294
GO:0061630 : ubiquitin protein ligase activity	13	0.020578947	2.549019608
GO:0005604 : basement membrane	5	0.021742386	0.980392157
GO:0000932 : P-body	6	0.023815078	1.176470588
GO:0007155 : cell adhesion	11	0.025498977	2.156862745
GO:0009986 : cell surface	12	0.030701127	2.352941176
GO:0008104 : protein localization	4	0.031916591	0.784313725
GO:0006590 : thyroid hormone	3	0.033444278	0.588235294

generation			
GO:0000164 : protein phosphatase type 1 complex	3	0.036210592	0.588235294
GO:0002040 : sprouting angiogenesis	3	0.038447746	0.588235294
GO:0015035 : protein-disulfide reductase activity	3	0.039137862	0.588235294
GO:0043547 : positive regulation of GTPase activity	6	0.039247378	1.176470588
GO:0001701 : in utero embryonic development	5	0.041411253	0.980392157
GO:1903078 : positive regulation of protein localization to plasma membrane	3	0.043715324	0.588235294
GO:0043353 : enucleate erythrocyte differentiation	2	0.044496918	0.392156863
GO:0060335 : positive regulation of type II interferon-mediated signaling pathway	2	0.044496918	0.392156863
GO:0000209 : protein polyubiquitination	6	0.044623419	1.176470588
GO:0016972 : thiol oxidase activity	2	0.04492781	0.392156863
GO:0043273 : CTPase activity	2	0.04492781	0.392156863
GO:0140785 : amino acid sensor activity	2	0.04492781	0.392156863
GO:0005903 : brush border	3	0.046410948	0.588235294
GO:0006470 : protein dephosphorylation	7	0.047244067	1.37254902
GO:0007275 : multicellular organism development	8	0.047313986	1.568627451
GO:0019901 : protein kinase binding	9	0.048236881	1.764705882
GO:0007162 : negative regulation of cell adhesion	3	0.049232652	0.588235294
GO:2000134 : negative regulation of G1/S transition of mitotic cell cycle	3	0.049232652	0.588235294

GO:0030324 : lung development	3	0.049232652	0.588235294

Table S3. GO enrichment analysis results of DEGs down-regulated in liver tissue.

GO Term	Count	p-value	Gene Ratio%
GO:0005737 : cytoplasm	217	0.0000000207	22.39422085
GO:0042802 : identical protein binding	40	0.0000000928	4.127966976
GO:0005739 : mitochondrion	63	0.0000019700	6.501547988
GO:0005634 : nucleus	229	0.0000068600	23.63261094
GO:0005654 : nucleoplasm	64	0.0000090700	6.604747162
GO:0008137 : NADH dehydrogenase (ubiquinone) activity	7	0.0000162000	0.722394221
GO:0005515 : protein binding	128	0.0001450000	13.20949432
GO:0038061 : non-canonical NF-kappaB signal transduction	5	0.0006610000	0.515995872
GO:0045271 : respiratory chain complex I	6	0.0006730000	0.619195046
GO:0016607 : nuclear speck	17	0.0006970000	1.754385965
GO:0000287 : magnesium ion binding	16	0.0008410000	1.651186791
GO:0045893 : positive regulation of DNA-templated transcription	18	0.001008306	1.857585139
GO:0006120 : mitochondrial electron transport, NADH to ubiquinone	6	0.001487126	0.619195046
GO:0005829 : cytosol	98	0.001611451	10.11351909
GO:0045944 : positive regulation of transcription by RNA polymerase II	30	0.001665506	3.095975232
GO:0005524 : ATP binding	83	0.001957748	8.565531476
GO:0042773 : ATP synthesis coupled electron transport	4	0.00195876	0.412796698
GO:0004713 : protein tyrosine kinase	6	0.002954306	0.619195046

activity			
GO:0008380 : RNA splicing	11	0.003081907	1.135190918
GO:0006468 : protein phosphorylation	30	0.003626111	3.095975232
GO:0000045 : autophagosome assembly	8	0.003914324	0.825593395
GO:0032981 : mitochondrial respiratory chain complex I assembly	7	0.004175085	0.722394221
GO:0003723 : RNA binding	49	0.004327494	5.056759546
GO:0003954 : NADH dehydrogenase activity	4	0.00467442	0.412796698
GO:0004129 : cytochrome-c oxidase activity	3	0.004815882	0.309597523
GO:0007249 : canonical NF-kappaB signal transduction	6	0.006186555	0.619195046
GO:0005681 : spliceosomal complex	10	0.006514561	1.031991744
GO:0071004 : U2-type prespliceosome	5	0.006881881	0.515995872
GO:0004674 : protein serine/threonine kinase activity	22	0.007274117	2.270381837
GO:0043122 : regulation of canonical NF-kappaB signal transduction	5	0.007291707	0.515995872
GO:0043484 : regulation of RNA splicing	6	0.008189006	0.619195046
GO:0006397 : mRNA processing	13	0.008734367	1.341589267
GO:0015990 : electron transport coupled proton transport	3	0.009027903	0.309597523
GO:0031119 : tRNA pseudouridine synthesis	3	0.009027903	0.309597523
GO:0043139 : 5'-3' DNA helicase activity	3	0.009372309	0.309597523
GO:0015143 : urate transmembrane transporter activity	3	0.009372309	0.309597523

GO:0034244 : negative regulation of transcription elongation by RNA polymerase II	4	0.010604298	0.412796698
GO:0046872 : metal ion binding	94	0.012253979	9.700722394
GO:0005811 : lipid droplet	8	0.013448983	0.825593395
GO:0005813 : centrosome	22	0.013879927	2.270381837
GO:0035631 : CD40 receptor complex	3	0.014189508	0.309597523
GO:0009982 : pseudouridine synthase activity	4	0.01740676	0.412796698
GO:0002020 : protease binding	6	0.018104169	0.619195046
GO:0004622 : lysophospholipase activity	4	0.021113017	0.412796698
GO:0042542 : response to hydrogen peroxide	3	0.021398679	0.309597523
GO:0019509 : L-methionine salvage from methylthioadenosine	3	0.021398679	0.309597523
GO:0005543 : phospholipid binding	8	0.021715973	0.825593395
GO:0019904 : protein domain specific binding	7	0.021786963	0.722394221
GO:0005685 : U1 snRNP	5	0.022723158	0.515995872
GO:0050852 : T cell receptor signaling pathway	7	0.023563288	0.722394221
GO:0030163 : protein catabolic process	4	0.023981229	0.412796698
GO:0042254 : ribosome biogenesis	6	0.027398315	0.619195046
GO:0001701 : in utero embryonic development	7	0.027495112	0.722394221
GO:0032021 : NELF complex	3	0.028281659	0.309597523
GO:0010890 : positive regulation of triglyceride storage	3	0.029174647	0.309597523

GO:0003678 : DNA helicase activity	4	0.029715483	0.412796698
GO:0042981 : regulation of apoptotic process	10	0.030138961	1.031991744
GO:0015031 : protein transport	18	0.030928501	1.857585139
GO:0000978 : RNA polymerase II cis-regulatory region sequence-specific DNA binding	60	0.032437872	6.191950464
GO:0019915 : lipid storage	4	0.032950259	0.412796698
GO:0006506 : GPI anchor biosynthetic process	5	0.033572137	0.515995872
GO:0006364 : rRNA processing	8	0.033674737	0.825593395
GO:0001825 : blastocyst formation	3	0.037885726	0.309597523
GO:0000423 : mitophagy	4	0.0379996	0.412796698
GO:0005762 : mitochondrial large ribosomal subunit	6	0.039096479	0.619195046
GO:0005743 : mitochondrial inner membrane	21	0.039747568	2.167182663
GO:0004521 : RNA endonuclease activity	4	0.03988911	0.412796698
GO:0044183 : protein folding chaperone	4	0.03988911	0.412796698
GO:0016301 : kinase activity	5	0.043550366	0.515995872
GO:0003682 : chromatin binding	17	0.044105919	1.754385965
GO:0005096 : GTPase activator activity	15	0.046631215	1.547987616
GO:0034389 : lipid droplet organization	3	0.047445192	0.309597523
GO:0006309 : apoptotic DNA fragmentation	3	0.047445192	0.309597523
GO:0016818 : hydrolase activity, acting on acid anhydrides, in phosphorus-containing anhydrides	3	0.049128245	0.309597523

GO:0097191 : extrinsic apoptotic signaling pathway	4	0.049200424	0.412796698

Table S4. KEGG enrichment analysis results of DEGs up-regulated in liver tissue.

Term	Count	*p*-value	%
oas05165:Human papillomavirus infection	20	0.002580026	3.921568627
oas00230:Purine metabolism	11	0.002851761	2.156862745
oas04141:Protein processing in endoplasmic reticulum	13	0.002940401	2.549019608
oas04512:ECM-receptor interaction	8	0.009935215	1.568627451
oas04514:Cell adhesion molecules	11	0.010070101	2.156862745
oas04620:Toll-like receptor signaling pathway	9	0.012899525	1.764705882
oas04151:PI3K-Akt signaling pathway	19	0.015004891	3.725490196
oas04510:Focal adhesion	12	0.019240236	2.352941176
oas04015:Rap1 signaling pathway	12	0.027053999	2.352941176
oas05200:Pathways in cancer	24	0.028374249	4.705882353
oas04145:Phagosome	10	0.032290304	1.960784314
oas04060:Cytokine-cytokine receptor interaction	15	0.057740861	2.941176471
oas05205:Proteoglycans in cancer	11	0.059849006	2.156862745
oas04148:Efferocytosis	9	0.069893774	1.764705882
oas05146:Amoebiasis	7	0.079152661	1.37254902
oas04142:Lysosome	8	0.0793194	1.568627451
oas04022:cGMP-PKG signaling pathway	9	0.081972224	1.764705882
oas04540:Gap junction	6	0.085013135	1.176470588

oas04927:Cortisol synthesis and secretion	5	0.096409548	0.980392157

Table S5. KEGG enrichment analysis results of DEGs down-regulated in liver tissue.

Term	Count	*p*-value	%
oas01100:Metabolic pathways	116	0.000000479	11.97110423
oas05208:Chemical carcinogenesis - reactive oxygen species	23	0.005069489	2.373581011
oas01232:Nucleotide metabolism	10	0.01268466	1.031991744
oas03083:Polycomb repressive complex	10	0.016733172	1.031991744
oas04623:Cytosolic DNA-sensing pathway	10	0.020337507	1.031991744
oas04714:Thermogenesis	21	0.024561351	2.167182663
oas00190:Oxidative phosphorylation	16	0.025224544	1.651186791
oas05014:Amyotrophic lateral sclerosis	29	0.0309484	2.992776058
oas05012:Parkinson disease	23	0.034198875	2.373581011
oas05135:Yersinia infection	13	0.036209086	1.341589267
oas00030:Pentose phosphate pathway	5	0.044866938	0.515995872
oas00563:Glycosylphosphatidylinositol (GPI)-anchor biosynthesis	5	0.044866938	0.515995872
oas01200:Carbon metabolism	11	0.045286691	1.135190918
oas04010:MAPK signaling pathway	21	0.047856153	2.167182663
oas03420:Nucleotide excision repair	7	0.053277938	0.722394221
oas04657:IL-17 signaling pathway	9	0.056189696	0.92879257
oas00051:Fructose and mannose metabolism	5	0.059761241	0.515995872
oas04146:Peroxisome	8	0.074625866	0.825593395
oas05016:Huntington disease	23	0.076245608	2.373581011

oas04723:Retrograde endocannabinoid signaling	12	0.083635184	1.238390093
oas05010:Alzheimer disease	27	0.08923836	2.786377709
oas01240:Biosynthesis of cofactors	12	0.089543419	1.238390093
oas04210:Apoptosis	12	0.095700309	1.238390093

Table S6. GO enrichment results of the MEblue in WGCNA results.

GO Term	Count	p-value	Gene Ratio%
GO:0030198 : extracellular matrix organization	16	0.0000020400	2.18579235
GO:0001666 : response to hypoxia	9	0.0000145000	1.229508197
GO:0003713 : transcription coactivator activity	16	0.0000199000	2.18579235
GO:0005515 : protein binding	106	0.0000823000	14.48087432
GO:0006695 : cholesterol biosynthetic process	7	0.0000868000	0.956284153
GO:0005604 : basement membrane	9	0.0001070000	1.229508197
GO:0062023 : collagen-containing extracellular matrix	15	0.0001160000	2.049180328
GO:0045944 : positive regulation of transcription by RNA polymerase II	27	0.0006100000	3.68852459
GO:0043066 : negative regulation of apoptotic process	15	0.0008310000	2.049180328
GO:0016324 : apical plasma membrane	16	0.001033224	2.18579235
GO:0043235 : receptor complex	12	0.002133473	1.639344262
GO:0001525 : angiogenesis	9	0.004057412	1.229508197
GO:0005829 : cytosol	77	0.004111731	10.51912568
GO:0005794 : Golgi apparatus	26	0.00562573	3.551912568

GO:0030020 : extracellular matrix structural constituent conferring tensile strength	5	0.00663903	0.683060109
GO:0030574 : collagen catabolic process	5	0.007728186	0.683060109
GO:0008285 : negative regulation of cell population proliferation	9	0.008227934	1.229508197
GO:0005201 : extracellular matrix structural constituent	6	0.010588259	0.819672131
GO:0008305 : integrin complex	5	0.011100691	0.683060109
GO:0042730 : fibrinolysis	4	0.011169902	0.546448087
GO:0006915 : apoptotic process	14	0.013035898	1.912568306
GO:0010508 : positive regulation of autophagy	5	0.013121562	0.683060109
GO:0005923 : bicellular tight junction	8	0.013355537	1.092896175
GO:0048008 : platelet-derived growth factor receptor signaling pathway	4	0.013425244	0.546448087
GO:0042803 : protein homodimerization activity	14	0.013979348	1.912568306
GO:0010629 : negative regulation of gene expression	7	0.014224437	0.956284153
GO:0045893 : positive regulation of DNA-templated transcription	13	0.014511883	1.775956284
GO:0007249 : canonical NF-kappaB signal transduction	5	0.014765443	0.683060109
GO:0032991 : protein-containing complex	9	0.016675919	1.229508197
GO:0043065 : positive regulation of apoptotic process	10	0.017503191	1.366120219
GO:0033627 : cell adhesion mediated by integrin	5	0.018429081	0.683060109
GO:0071300 : cellular response to retinoic	4	0.01865826	0.546448087

acid			
GO:0007275 : multicellular organism development	11	0.019273457	1.50273224
GO:0044877 : protein-containing complex binding	7	0.020151202	0.956284153
GO:0005509 : calcium ion binding	33	0.020288883	4.508196721
GO:0004222 : metalloendopeptidase activity	10	0.021008268	1.366120219
GO:0016323 : basolateral plasma membrane	10	0.022670398	1.366120219
GO:0007155 : cell adhesion	14	0.023096123	1.912568306
GO:0005777 : peroxisome	7	0.023852305	0.956284153
GO:0045202 : synapse	16	0.024774769	2.18579235
GO:0000124 : SAGA complex	4	0.024935294	0.546448087
GO:0016209 : antioxidant activity	3	0.025206671	0.409836066
GO:0000932 : P-body	7	0.026830735	0.956284153
GO:0016607 : nuclear speck	11	0.02805467	1.50273224
GO:0097191 : extrinsic apoptotic signaling pathway	4	0.028336115	0.546448087
GO:0000139 : Golgi membrane	19	0.02837234	2.595628415
GO:0009653 : anatomical structure morphogenesis	7	0.029433127	0.956284153
GO:0005654 : nucleoplasm	39	0.031048191	5.327868852
GO:0035987 : endodermal cell differentiation	3	0.031778062	0.409836066
GO:0016787 : hydrolase activity	12	0.032725191	1.639344262
GO:0005778 : peroxisomal membrane	5	0.032896797	0.683060109

GO:0006468 : protein phosphorylation	22	0.033593209	3.005464481
GO:0036464 : cytoplasmic ribonucleoprotein granule	4	0.035488303	0.546448087
GO:0010628 : positive regulation of gene expression	10	0.037866041	1.366120219
GO:0016477 : cell migration	11	0.040067211	1.50273224
GO:0005319 : lipid transporter activity	4	0.040113963	0.546448087
GO:0002062 : chondrocyte differentiation	4	0.040195187	0.546448087
GO:0045727 : positive regulation of translation	4	0.040195187	0.546448087
GO:0007160 : cell-matrix adhesion	6	0.040300832	0.819672131
GO:0007229 : integrin-mediated signaling pathway	6	0.040300832	0.819672131
GO:0030335 : positive regulation of cell migration	8	0.04359861	1.092896175
GO:0005739 : mitochondrion	36	0.045630314	4.918032787
GO:0006607 : NLS-bearing protein import into nucleus	3	0.046545525	0.409836066
GO:0005912 : adherens junction	9	0.047517505	1.229508197
GO:0004197 : cysteine-type endopeptidase activity	6	0.048109933	0.819672131
GO:0042632 : cholesterol homeostasis	5	0.048140699	0.683060109
GO:0007417 : central nervous system development	6	0.048245655	0.819672131
GO:0031593 : polyubiquitin modification-dependent protein binding	4	0.049183292	0.546448087

Table S7. KEGG enrichment results of the MEblue in WGCNA results.

**Term**	**Count**	***p*-value**	**%**

oas04514:Cell adhesion molecules	20	0.0000364	2.732240437
oas04512:ECM-receptor interaction	12	0.001286904	1.639344262
oas00100:Steroid biosynthesis	6	0.002052803	0.819672131
oas04146:Peroxisome	10	0.007219324	1.366120219
oas04520:Adherens junction	11	0.007690482	1.50273224
oas04933:AGE-RAGE signaling pathway in diabetic complications	11	0.009410428	1.50273224
oas04670:Leukocyte transendothelial migration	12	0.011306386	1.639344262
oas01100:Metabolic pathways	86	0.028181286	11.74863388
oas04820:Cytoskeleton in muscle cells	17	0.033111977	2.322404372
oas04658:Th1 and Th2 cell differentiation	9	0.036455984	1.229508197
oas05215:Prostate cancer	9	0.049779304	1.229508197
oas04979:Cholesterol metabolism	6	0.060644571	0.819672131
oas04510:Focal adhesion	14	0.082983361	1.912568306
oas04151:PI3K-Akt signaling pathway	23	0.087001288	3.142076503

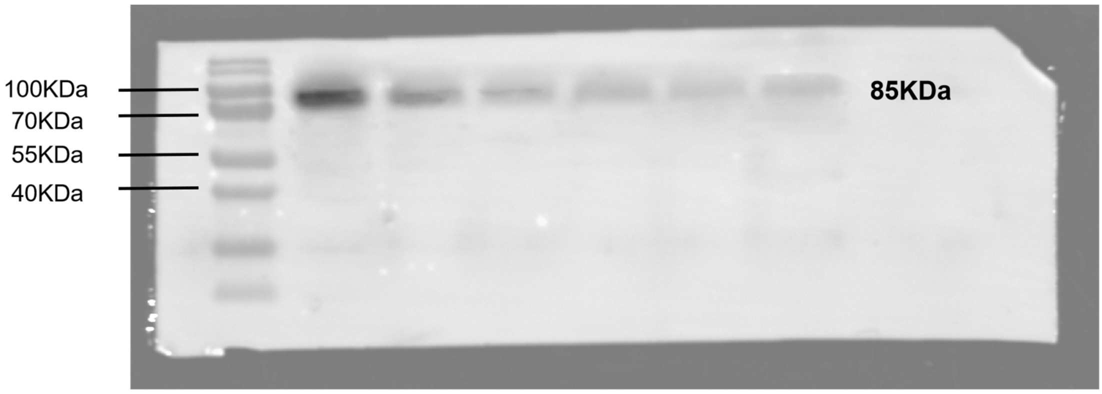

Figure S1. Original uncropped Western blot membrane for p-PI3K, corresponding to the assembled blot shown in Figure 10.

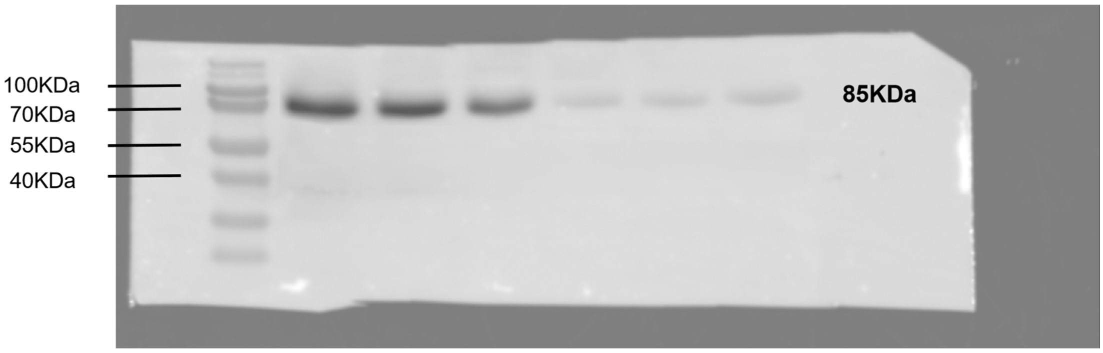

Figure S2. Original uncropped Western blot membrane for PI3K, corresponding to the assembled blot shown in Figure 10.

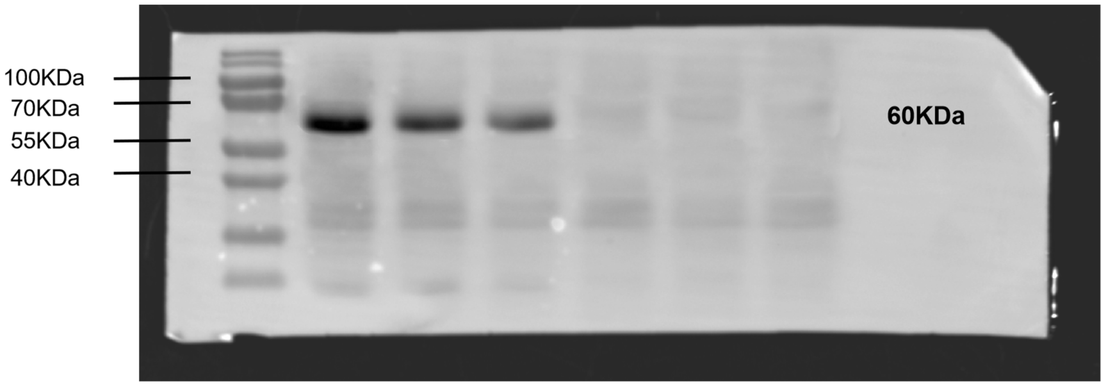

Figure S3. Original uncropped Western blot membrane for p-AKT, corresponding to the assembled blot shown in Figure 10.

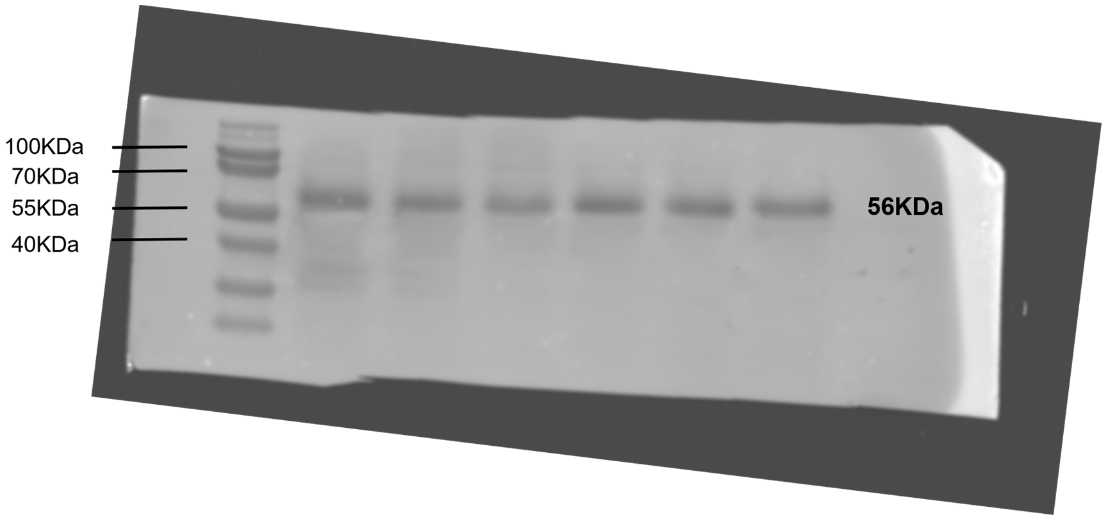

Figure S4. Original uncropped Western blot membrane for AKT, corresponding to the assembled blot shown in Figure 10.

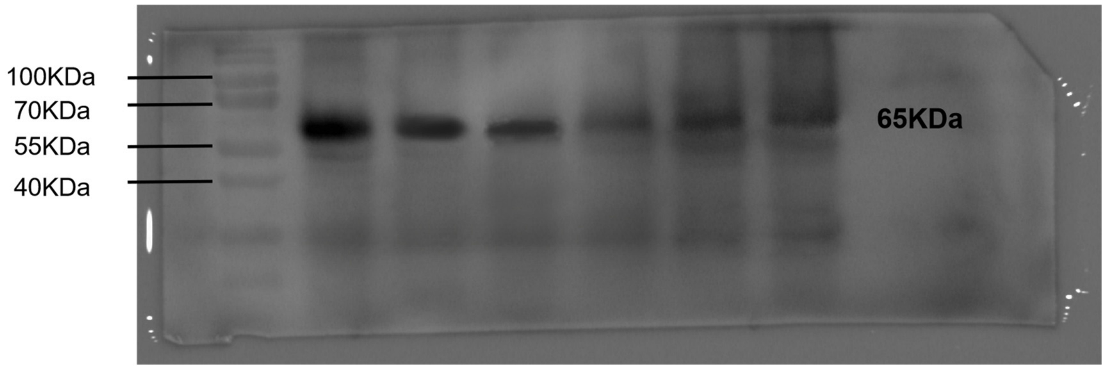

Figure S5. Original uncropped Western blot membrane for p-NFκB, corresponding to the assembled blot shown in Figure 10

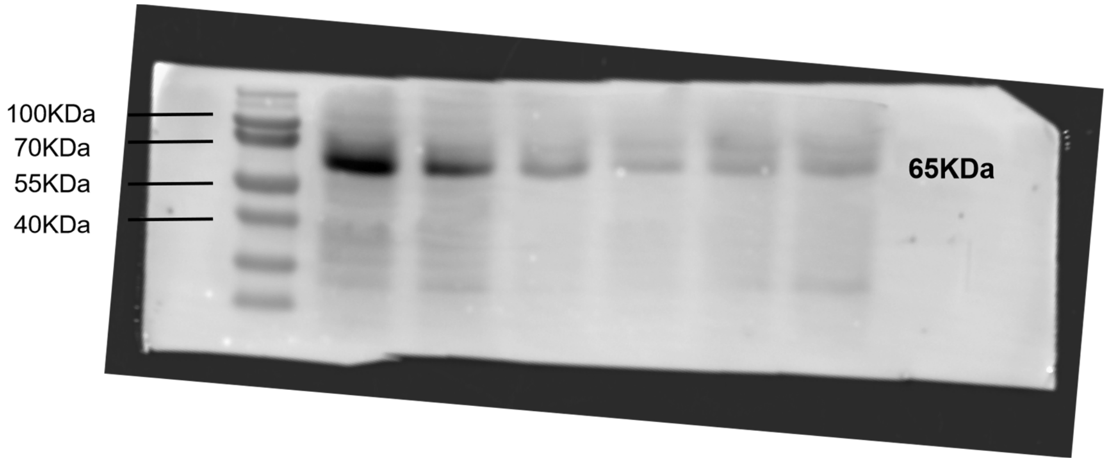

.**Figure S6.** Original uncropped Western blot membrane for NFκB, corresponding to the assembled blot shown in Figure 10

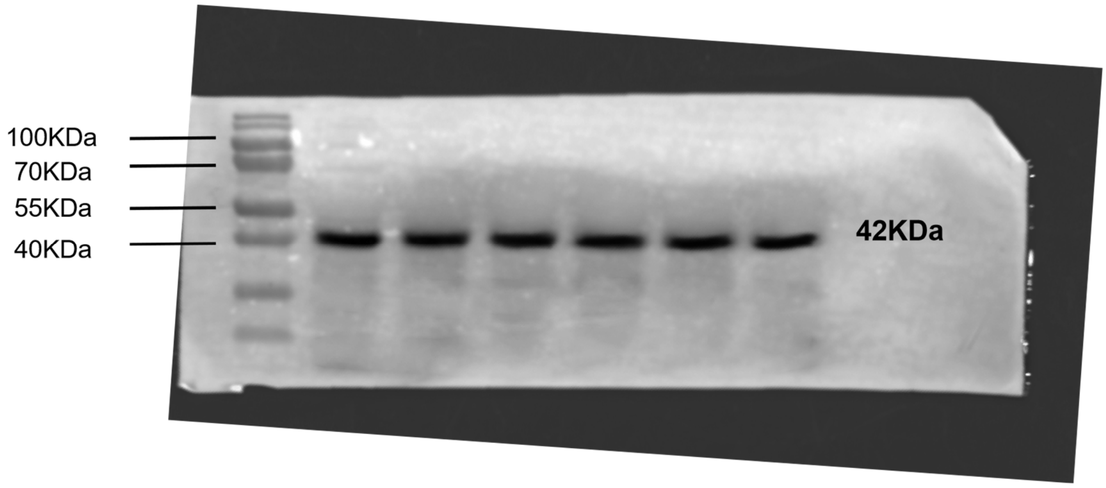

Figure S7. Original uncropped membrane showing the β-actin internal control used for normalization of the phosphorylated proteins in Figure 10.

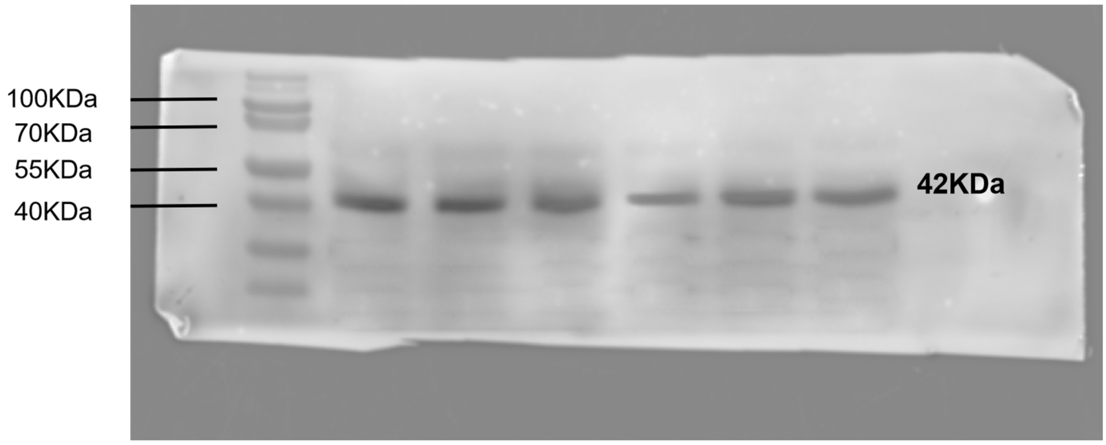

Figure S8. Original uncropped membrane showing the β-actin internal control used for normalization of the total proteins in Figure 10.
